# Efficiency of different air filter types for pig facilities at laboratory scale

**DOI:** 10.1371/journal.pone.0186558

**Published:** 2017-10-13

**Authors:** Cindy Wenke, Janina Pospiech, Tobias Reutter, Uwe Truyen, Stephanie Speck

**Affiliations:** 1 Institute of Animal Hygiene and Veterinary Public Health, Leipzig, Germany; 2 REVENTA® GmbH, Horstmar, Germany; Sun Yat-Sen University, CHINA

## Abstract

Air filtration has been shown to be efficient in reducing pathogen burden in circulating air. We determined at laboratory scale the retention efficiency of different air filter types either composed of a prefilter (EU class G4) and a secondary fiberglass filter (EU class F9) or consisting of a filter mat (EU class M6 and F8-9). Four filter prototypes were tested for their capability to remove aerosol containing equine arteritis virus (EAV), porcine reproductive and respiratory syndrome virus (PRRSV), bovine enterovirus 1 (BEV), *Actinobacillus pleuropneumoniae* (*APP*), and *Staphylococcus (S*.*) aureus* from air. Depending on the filter prototype and utilisation, the airflow was set at 1,800 m^3^/h (combination of upstream prefilter and fiberglass filter) or 80 m^3^/h (filter mat). The pathogens were aerosolized and their concentration was determined in front of and behind the filter by culture or quantitative real-time RT-PCR. Furthermore, survival of the pathogens over time in the filter material was determined. Bacteria were most efficiently filtered with a reduction rate of up to 99.9% depending on the filter used. An approximately 98% reduction was achieved for the viruses tested. Viability or infectivity of *APP* or PRRSV in the filter material decreased below the detection limit after 4 h and 24 h, respectively, whereas *S*. *aureus* was still culturable after 4 weeks. Our results demonstrate that pathogens can efficiently be reduced by air filtration. Consequently, air filtration combined with other strict biosecurity measures markedly reduces the risk of introducing airborne transmitted pathogens to animal facilities. In addition, air filtration might be useful in reducing bioaerosols within a pig barn, hence improving respiratory health of pigs.

## Introduction

Biosecurity measures are of utmost importance for a high standard in animal husbandry. Minimizing the risk of the introduction of pathogens into livestock herds is crucial to maintain a high health status. Routine measures include the use of protective clothing, quarantine procedures, instructions for cleaning and disinfection and others. Studies performed in the United States have shown that supply air filtration in pig facilities can prevent the entry of airborne pathogens such as the porcine reproductive and respiratory syndrome virus (PRRSV) and *Mycoplasma* (*M*.) *hyopneumoniae* ([1–3]. The spread via aerosols is well documented for PRRSV and *M*. *hyopneumoniae* with a long-distance airborne transport of up to 10 km [4,5]. Investigations on American farms equipped with air filtration revealed high efficacy in reducing the number of PRRSV outbreaks. The incidence of new PRRSV infections in breeding herds housed without air filtration was reported to be eight times higher compared to filtered farms [6]. However, commercial swine buildings with supply air filtration are still not a standard.

Besides the capacity of air filters to protect herds against airborne transmission from outside their ability to reduce bioaerosol droplets containing potential pathogens as well as dust in circulating air is also of interest. Pathogens are mostly associated with dust particles [7] and a high airborne germ load may lead to faster spread of the pathogens among herds. Moreover, damages of the respiratory tract, caused by high dust pollution inside the stable, can increase susceptibility to infections with certain pathogens [8].

There are air filters of different classification available and nomenclature varies according to European or American standards. In principle, Coarse Filters (pre filter, EU class G) [9], Fine Filters (medium filter, EU class M and F) [9], High Efficiency Particulate Air (HEPA) filters (EU class H) [10], and Ultra Low Penetration Air (ULPA) filters (EU class U) [10] exist. HEPA and ULPA filters are commonly used to clear air in pharmaceutical industry and hospital settings as well as of microbiological laboratories. The costs of installing HEPA filters in a commercial swine facility were calculated to be approximately $ 1,500–2,000 USD per boar/sow [11]. Hence, a cost-efficient filter, easy to implement in an already existing ventilation plant with long service intervals would be of great interest.

The purpose of this study was to determine the air filtration efficiency of four different commercially available air filters for selected viruses and bacteria at laboratory scale in order to choose the most appropriate and affordable filter for pig facilities.

## Materials and methods

### Filter prototypes and test facility setup

**Fig 1:**
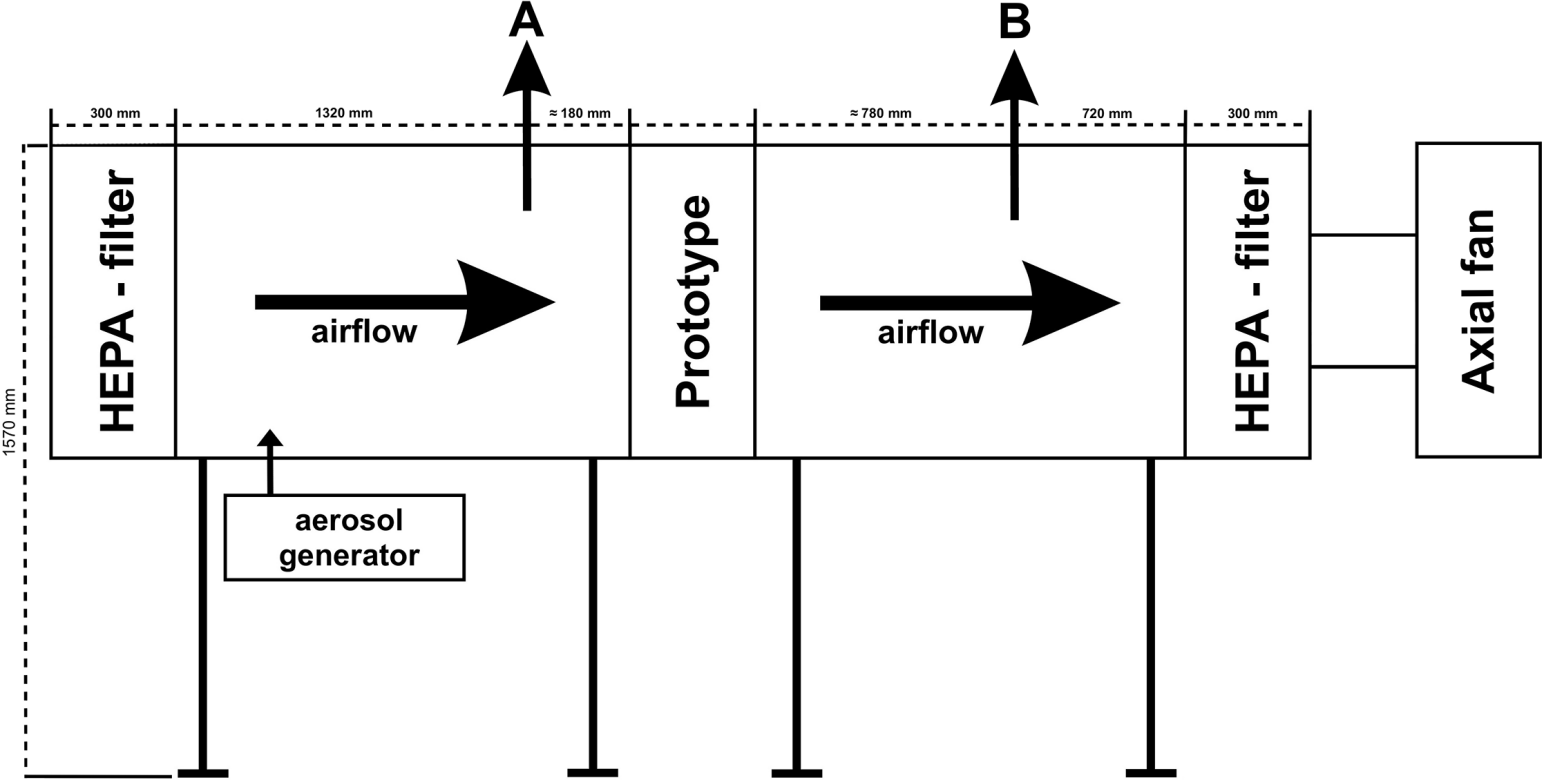
Schematic design of the test chamber. A: first sampling point in front of the filter, B: second sampling point behind the filter. Dimensions are given in mm.

Four mechanical filters with different levels of filtration efficiency were tested. A description of the four filter prototypes is given in Table 1. Briefly, prototypes 1 and 2 were composed of a prefilter and a secondary filter whereas prototypes 3 and 4 consisted of a filter wool mat. The secondary filters of both, prototype 1 and 2, had been determined to be >95% efficient at removing particles equal to or greater than 0.4 μm in diameter. Prototypes 1 and 2 are suitable for positive and negative pressure ventilation systems whereas prototypes 3 and 4 are designed only for negative pressure ventilation systems. Filter retention efficiency for selected pathogens was tested in a specific test chamber. It consisted of two identical elements, 150 cm in length and 59 cm in width and height, separated by the respective air filter prototype (Fig 1), dividing the he test chamber into a crude gas side (inlet) and a clean gas side (outlet). The pathogens were aerosolized by an ATM 230 (Topas GmbH, Dresden, Germany) and supplied into the chamber via a flexible tube using gauge pressure. Air samples were collected over 20 min at a flow rate of 550 l/h in front of (A) and behind (B) the tested filter prototypes (Fig 1). An axial fan ensured a continuous airflow. HEPA filters (class E13) at the air inlet and outlet assured clean air intake and a virus-free outlet air. Tests were performed at different volume flow rates depending on the field of filter application in a subsequent case-control study at a pig fattening facility. Prototypes 1 and 2 were tested as candidates for a high velocity ventilation system. According to the structural conditions at the pig facility these prototypes were tested at a volume flow rate of 1,800 m^3^/h. Prototype 3 and 4 were intended to use in combination with a diffused air ceiling and were thus tested at 80 m^3^/h. Consequently a comparison of the filter retention efficacy between filter 1 and 2 as well as between filter 3 and 4 was performed. Preliminary tests were conducted with each filter prototype using Equine arteritis virus (EAV) and *Staphylococcus* (*S*.) *aureus* exemplarily in order to choose the most efficient filter from group 1 (filter 1 compared to filter 2) and group 2 (filter 3 compared to filter 4) for further testing.

**Table 1:** Technical information of the filter prototypes.

Characteristic	Filter 1		Filter 2		Filter 3	Filter 4
	Prefilter	Secondary filter	Prefilter	Secondary filter		
**Description**	Panel filter	Compact filter	Panel filter	Compact filter	Filter mat	Filter wool with glass fiber
**Filter matter**	Polyester	Glass fiber	Synthetic-organic fiber	Glass fiber	Polyester	Glass wool
	Thickness 3 mm	Thickness 0.55 mm	Thickness 0.2 mm	Thickness 0.6 mm	Thickness 25 mm	Thickness 2 x 40 mm
	Base Weight 200 g/m^2^	Base Weight 68 g/m^2^	Base Weight 145 g/m^2^	Base Weight 79 g/m^2^	Base Weight 650 g/m^2^	Base Weight 1,140 g/m^2^
**Size (mm)**	592 x 592 x 48	592 x 592 x 292	595 x 595 x 48	593 x 593 x 292	1,200 x 1,200	1,200 x 1,200
**Filter surface**	1.2 m^2^	18.8 m^2^	1.1 m^2^	18 m^2^	1.35 m^2^	1.35 m^2^
**Initial pressure loss**	70 Pa at 2.7 m/s	110 Pa at 2.7 m/s	75 Pa at 2.7 m/s	105 Pa at 2.7 m/s	20 Pa at 0.1 m/s	50 Pa at 0.1 m/s
**EU EN 779 class [9]**	G4	F9	G4	F9	M6	approx. F8–F9*
**US ASHRAE standard 52.2–2007 [12]**	MERV 6–8	MERV 16	MERV 6–8	MERV 16	MERV 9–13	MERV 14–16

MERV-minimum efficiency reported value

* not tested according to EN 779 [9]

### Choosing pathogens and preparation of cultures

Three viruses and three bacterial species were chosen for the experiments. EAV (strain Bucyrus) was used as a representative for viruses in preliminary tests with all four filter prototypes. This virus is closely related to PRRSV [13] and routinely maintained in our laboratory at high titers. It was grown in Vero B4 cells (CCLV RIE 1146) using Iscove’s Modified Dulbecco’s Medium (Life Technologies GmbH, Darmstadt, Germany) mixed 1:2 with Ham’s F12 Nutrient Mixture (Life Technologies GmbH) + 5% fetal calf serum (FCS; Sigma-Aldrich Chemie GmbH, Schnelldorf, Germany) at 37°C. We chose PRRSV because of its economic impact in swine industry [14]. Moreover, several studies had already demonstrated the efficacy of some air filter methods in reducing PRRSV under experimental conditions [1,15–17,6]. Further experiments were performed using bovine enterovirus 1 (BEV, strain LCR-4) as a surrogate for foot-and-mouth disease virus (FMDV). Both are small non-enveloped RNA-viruses and belong to the family *Picornaviridae* [18]. Foot-and-mouth disease still remains an important economic concern in livestock production, especially in swine and cattle. FMDV is a highly contagious virus and evidence of long-distance airborne transport up to 250 km has been reported [19,20]. PRRSV (PRRS® Porcilis EU live attenuated vaccine, MSD Animal Health, Unterschleissheim, Germany) and BEV were grown in MARC-145 (CCLV RIE 277) and MDBK cells (CCLV RIE 261), respectively, both maintained at 37°C in MEM Hank`s salts (with L-Glutamine; Life Technologies GmbH) and MEM Earle`s salts (with L-Glutamine; Life Technologies GmbH) supplemented with non-essential amino acids (Life Technologies GmbH), sodium pyruvate (Life Technologies GmbH) and 10% FCS (Sigma-Aldrich Chemie GmbH). All cell lines were obtained from the Collection of Cell Lines in Veterinary Medicine (CCLV), Friedrich-Loeffler-Institute, Greifswald—Insel Riems, Germany. Pooled cell culture supernatants of each virus were cleared by low-speed centrifugation and stored in 50 ml-aliquots at -80°C until further use. Virus suspensions had a titer of 10^6.7^–10^7.6^ tissue culture infectious dose (TCID)_50_/ml (EAV), 10^5.3^–10^5.8^ TCID_50_/ml (PRRSV), and 10^5.3^–10^5.6^ TCID_50_/ml (BEV).

As a representative for Gram-positive bacteria, *S*. *aureus* (strain DSM 799) was chosen for the preliminary tests. Staphylococci and other Gram-positive bacteria constitute 80% of the total airborne germs inside livestock housings [21]. Moreover, Methicillin-resistant *S*. *aureus* (MRSA) was found in air samples from pig barns in high numbers. MRSA is bound to and spread via dust particles [22]. *S*. *aureus* cultures were grown in Tryptic Soy Broth (Carl Roth GmbH + Co. KG, Karlsruhe, Germany) at 37°C over night to reach 10^8^−10^9^ colony-forming units (cfu)/ml and were subsequently used for filter experiments. *Actinobacillus pleuropneumoniae* (*APP*) and mycoplasma cause various diseases in swine. *APP* is known to be transmitted over only short distances [23,24] whereas mycoplasma are able to spread by aerosol over long distances [5]. *APP* (type strain, DSM 13472) was cultured in PPLO-broth (Acumedia, Lansing, USA) supplemented with nicotinamide adenine dinucleotide (10 mg/l; Carl Roth GmbH + Co. KG) at 37°C for 18 h resulting in 6 x 10^8^ cfu/ml. *APP* cultures were likewise subsequently used. *Mycoplasma* (*M*.) *hyorhinis* (type strain BTS-7, DSM 25591) was grown in liquid Friis-medium (according to European Pharmakopöe) at 37°C to obtain 10^6^−10^7^ cfu/ml and was stored in 50 ml-aliquots at -80°C until experiments were performed. All bacterial strains were obtained from the Leibniz Institute DSMZ-German Collection of Microorganisms and Cell Cultures, Braunschweig, Germany. In a set of preceding tests, filter matters and the gelatin filter for air sampling were tested for toxicity for each cell line and bacterium.

### Experiments

Five replicates were performed with every filter and the respective pathogen. Filters were changed between the various pathogens. Pathogens were aerosolized by the Atomizer Aerosol Generator ATM 230 (Topas GmbH) filled with 50 ml of the respective pathogen culture. ATM 230 produces droplet aerosols with known properties according to the guideline VDI 3491 [25]. According to the manufacturer the ATM 230 warrants highly constant particle size distribution as well as particle concentration with high reproducibility and a high aerosol output. A particle impaction section removed coarse spray droplets resulting in a particle size distribution of 0.2 μm to 1 μm. HEPA-filtered compressed air of 5 bar (tests of prototype 1 and 2) and 3.5 bar (tests of prototype 3 and 4) was used to aerosolize pathogen suspensions.

Air was collected using an air sampler pump (Analyt-MTC GmbH, Müllheim, Germany) and water-soluble gelatin filters (Sartorius 12602-80-ALK, Sartorius AG, Göttingen, Germany) in front of (Fig 1A) and behind the filter prototype (Fig 1B). The airflow rate was set at 550 l/h and sampling was performed for 20 min. For determination of infectious virus particles and bacteria, each gelatin filter was dissolved at 37°C in 5 ml of the respective growth medium. Solutions were filtered through a 0.2 μm syringe filter prior to virus titration and virus titer was calculated with the formula of Spaermann and Kärber [26,27]. Each virus titration was repeated once.

Bacteria were enumerated by the spread-plate method. Samples were plated onto Tryptic Soy Agar (*S*. *aureus*; Carl Roth GmbH & Co. KG), Chocolate Agar with Vitox (*APP*; OXOID Deutschland GmbH, Wesel, Germany) or Friis agar (*M*. *hyorhinis*) in duplicates and incubated at 37°C, 5% CO_2_, as described before.

Filter retention was calculated according to the following equation:

$$Filter\;reduction\;(\%)=\frac{pathogen\;number\;behind\;the\;filter}{pathogen\;number\;in\;front\;of\;the\;filter}*100\;\%$$

In addition, viability of pathogens in culture residuals recovered from the Atomizer ATM 230 bowl was assessed.

### Quantitative real-time RT-PCR for the detection of BEV

As BEV turned out to be very susceptible for desiccation, we additionally investigated all samples by PCR in order to get a second estimate. All dissolved samples were subjected to RNA isolation by the RNeasy® Mini Kit (Qiagen, Hilden, Gemany) and were tested for the presence of virus RNA using the SuperScript III Platinum® One-Step Quantitative RT-PCR System (Life Technologies Corporation, Carlsbad, USA) according to the manufacturers’ instructions. Primers (BEV-5FL 5’-GCCGTGAATGCTGCTAATCC-3’, BEV-3FL 5'-GTAGTCTGTTCCGCCTCCACCT-3’) and probe (BEV-SON 5’-6FAM-CGCACAATCCAGTGTTGCTACGTCGTAAC-3’ BBQ) were adopted from our colleagues [28] with minor modifications.

### Survival of pathogens inside the filter matter

After each experiment the filter was boxed, labelled with the date of the experiment and the used pathogen and stored at room temperature (average +20°C). To monitor the viability and to verify a possible multiplication of the pathogens inside the filter matter, samples of each filter were taken at certain intervals (30 min, 60 min, 120 min, 240 min, 24 h, 48 h, 7 d and 4 weeks) after the experiment until growth was no longer recorded. Five pieces (each 1 cm x 1 cm) of the prefilter and the secondary filter were taken with a sterile scalpel and tweezers. Samples of the respective filter part were pooled and incubated in the appropriate culture medium for 10 minutes. Virus titration, bacterial culture and PCR were performed as described above.

### Data analysis

Statistical analysis was made with SPSS Statistic 22 (IBM Deutschland GmbH, Ehningen, Germany). For comparison of filter prototype 1 with prototype 2 as well as prototype 3 with prototype 4 the exact Wilcoxon-Mann-Whitney-U-test was used to evaluate whether a respective filter revealed better retention efficiency.

## Results

None of the four prototype filter matters revealed toxic activity against the cell lines and bacteria used. With regard to pathogen amount filled into the atomizer and pathogen count in the test chamber in front of the filter, a high loss of infectious particles was noticed for all trials irrespective of the pathogen used (Table 2). The four filter prototypes revealed different retention efficiencies with the various pathogens (Table 2). Due to filter composition a comparison of retention rates was possible only for prototype 1 and 2 as well as for prototype 3 and 4.

**Table 2:** Retention efficiency determined for the different filter prototypes.

Pathogen	Prototype	Pathogen amount			Reduction efficiency (%) ± standard deviation
		in culture suspension filled into the atomizer	in front of the filter	behind the filter	
EAV	1	10^7.5^ TCID_50_/ml	10^3.7^ TCID_50_/ml	10^2.1^ TCID_50_/ml	97.5 ± 1.19
	2	10^6.7^ TCID_50_/ml	10^3.6^ TCID_50_/ml	10^2.0^ TCID_50_/ml	97.5 ± 2.36
	3*	10^7.6^ TCID_50_/ml	10^5.0^ TCID_50_/ml	10^3.6^ TCID_50_/ml	96.0 ± 13.01
	4	10^7.3^ TCID_50_/ml	10^4.7^ TCID_50_/ml	10^2.8^ TCID_50_/ml	98.7 ± 1.26
*S*. *aureus*	1	6.2 x 10^8^ cfu/ml	5.4 x 10^5^ cfu/ml	7.2 x 10^3^ cfu/ml	98.6 ± 0.29
	2^#^	1.5 x 10^8^ cfu/ml	3.2 x 10^5^ cfu/ml	2.6 x 10^3^ cfu/ml	99.2 ± 0.21
	4	1.3 x 10^8^ cfu/ml	5.4 x 10^5^ cfu/ml	9.1 x 10^1^ cfu/ml	99.97 ± 0.07
PRRSV	1	10^5.8^ TCID_50_/ml	10^3.8^ TCID_50_/ml	10^2.1^ TCID_50_/ml	98.0 ± 1.05
	4	10^5.3^ TCID_50_/ml	10^2.9^ TCID_50_/ml	10^1.8^ TCID_50_/ml	92.1 ± 5.96
BEV	1	1.5 x 10^10^ copies/μl template^$^	5.6 x 10^6^ copies/μl template	1.9 x 10^5^ copies/μl template	96.0 ± 2.90
	4	1.5 x 10^10^ copies/μl template	1.2 x 10^8^ copies/μl template	1.5 x 10^6^ copies/μl template	98.7 ± 0.72
*APP*	1	3.8 x 10^8^ cfu/ml	2.8 x 10^3^ cfu/ml	1.6 x 10^2^ cfu/ml	95.2 ± 3.34
	4	3.5 x 10^8^ cfu/ml	6.7 x 10^4^ cfu/ml	6.5 x 10^1^ cfu/ml	99.9 ± 0.05
*M*. *hyorhinis*	1	3.6 x 10^6^ cfu/ml	0 cfu/ml	0 cfu/ml	na
		1.3 x 10^7^ cfu/ml	0 cfu/ml	0 cfu/ml	na

TCID—tissue culture infectious dose; cfu–colony-forming units; na–not applicable

*sorted out after the first experiments due to compaction of the filter matter

#excluded because of economic reasons

^$^determined by quantitative real-time RT-PCR

Preliminary tests with all four filters were done with EAV and *S*. *aureus*. Regarding EAV, there was no significant difference in retention rates determined for prototype 1 and 2 (p = 1.000). Prototype 3 revealed a slightly lower filter efficiency compared to prototype 4 (p = 0.286). Moreover, clogging of prototype 3 was noticed during the experiment. As a consequence, a step-wise adjustment at the frequency converter was necessary in order to keep the volume flow rate at 80 m^3^/h. Therefore, prototype 3 was excluded after the first experiments using EAV. Prototypes 1, 2 and 4 were further tested with *S*. *aureus* as a representative for bacteria. Compared to prototype 2, prototype 1 achieved a significant lower retention efficiency for *S*. *aureus* (p = 0.016). However, although prototype 2 revealed a reduction rate of >99% it was sorted out due to slightly higher maintenance costs. Prototype 4 filtered *S*. *aureus* to 99.97% from air.

All further tests using PRRSV, BEV, and *APP* were done with prototype 1 (two-part filter system, 1,800 m^3^/h, air velocity 1.4 m/s) and prototype 4 (filter wool, 80 m^3^/h, air velocity 0.06 m/s). Prototype 1 revealed the highest reduction rate for PRRSV (i.e. 98.0%) and the lowest for BEV (96.0%). Reduction efficiencies achieved for *S*. *aureus* and *APP* were 98.6% and 95.2%, respectively. Prototype 4, tested at a lower volume flow rate of 80 m^3^/h, revealed a nearly 100% reduction of *APP* but was less efficient for EAV, BEV and PRRSV (Table 2).

Experiments using *M*. *hyorhinis* failed. This might be explained by a low initial bacterial titer (10^6^−10^7^ cfu/ml) or by agglomeration of bacteria cells resulting in insufficient amounts of bacteria released into the test chamber (Table 2). Even the use of a magnetic stirrer and a drilled nozzle of the atomizer did not lead to success.

After 20 min of continuous aerosolization (i.e. the length of an experiment) a decrease in viability was only seen for PRRSV and APP. Viability of PRRSV decreased up to 75% whereas viability of APP was reduced by over 90%.

*S*. *aureus* stayed infectious for four weeks in both prefilter and secondary filter of prototype 1 but not in prototype 2 (Table 3). A 1000-fold decrease in cfu/ml was achieved within the first week of storage followed by another 100-fold reduction after 4 weeks. Viability also disappeared after four weeks in the glass wool of prototype 4. In contrast, *APP* stayed viable only up to 4 h in both filter parts of prototype 1 and was inconsistently recovered up to 4 h of storage from prototype 4. PRRSV was isolated from the secondary filter matter of prototype 1 and from prototype 4 lastly 24 h after the experiment had ended. BEV was not grown from any of the filter matters.

**Table 3:** Kinetics of infectivity in the filter material.

Pathogen	Prototype		Pathogen infectivity after selected points in time								
			0.5 h	1 h	2 h	4 h	24 h	48 h	1 w	4 w	6 m
*S*. *aureus*	1	PF	nd	nd	nd	nd	+	+	+	+	-
		SF	nd	nd	nd	nd	+	-	+	+	-
	2	PF	nd	nd	nd	nd	+	+	+	-	nd
		SF	nd	nd	nd	nd	+	+	+	-	nd
	4	glass wool	nd	nd	nd	nd	+	+	+	-	nd
PRRSV	1	PF	-	-	-	-	-	-	-	-	nd
		SF	+	+	+	+	+	-	-	-	nd
	4	glass wool	+	-	+	+	+	-	-	nd	nd
BEV	1	PF	-	-	-	-	-	-	nd	nd	nd
		SF	-	-	-	-	-	-	nd	nd	nd
	4	glass wool	-	-	-	-	-	-	nd	nd	nd
*APP*	1	PF	+	+	+	+	-	-	-	nd	nd
		SF	+	+	+	+	-	-	-	nd	nd
	4	glass wool	+	+	-	+	-	-	-	nd	nd

PF–Prefilter; SF—Secondary filter; nd-not done; h- hour; w-week; m-month; + bacterial /viral growth; − no bacterial /viral growth

## Discussion

The objective of this study was to evaluate and compare the efficiency of different air filter types tested at laboratory scale in an attempt to identify suitable candidates for air filtration in swine industry. This laboratory part was a prerequisite for a case-control study under field conditions performed subsequently (to be reported elsewhere). In Germany, filters for supply air are not commonly used in pig production although several studies demonstrated the ability to protect pig populations from airborne pathogen transmission by filtering fresh air [1,6,29,30]. In contrast, air filtration systems have already been implemented in numerous sow farms in pig dense areas in the Midwest of the US [31].

Prototypes 1 and 2 utilized in our study each consisted of an EU F9 (MERV 16) fiberglass filter combined to an upstream prefilter (EU G4, MERV 6–8). Both prototypes are suitable for positive and negative pressure ventilation systems but differ in prefilter media, thickness, and base weight (Table 1). When used in pig barns, the fiberglass filters need to be changed every two to three years. The prefilter increases the filter lifespan and reduces investment outlay. Prototypes 3 (EU M6) and 4 (EU F8-9) also differ regarding filter media, thickness, and base weight (Table 1) and are both only suitable for negative pressure ventilation systems. In the first preliminary experiments, air filters eliminated EAV to ≥96% from air. Although EAV is a small virus of approximately 0.06 μm in diameter all filter prototypes revealed results comparable to the average efficiency at 0.4 μm (i.e. >95%) determined by the manufacturer. Aerosol droplets produced by the ATM 230 Atomizer range from 0.2 μm up to 1.0 μm in size (according to the manufacturer’s information) and virus particles bound to these droplets are therefore easier to trap. This also reflects the situation at a pig barn as potential pathogens occur as bioaerosol droplets bound to moist, dust and other environmental components [32].

Overall, prototype 4 revealed highest pathogen reduction rates with the exception of tests using PRRSV. Compared to EAV PRRSV is similar in size and this would imply a similar reduction rate for both viruses which was achieved using prototype 1. However, results obtained for both viruses using prototype 4 varied markedly. Only marginal differences were seen between the five PRRSV-replicates hence a bias in test performance and analyses could be excluded. It has been described that the source of the raw material influences filter quality [32]. Prototype 4-experiments using EAV and PRRSV were done with a time-lag of five months and we had to reorder prototype 4. Therefore, minor differences in filter material of different lot numbers cannot fully excluded and might be a possible explanation for the different results.

Bacteria are much larger in size than viruses and the higher efficiency obtained using *S*. *aureus* and *APP* is much likely a matter of particle size. A 99.9%-efficiency was achieved using prototype 4, *APP* and *S*. *aureus*. Prototype 1 was less efficacious using *APP* although actinobacilli are larger in size (max. 0.5 x 1.4 μm) than staphylococci suggesting a similar or even higher filter retention rate compared to *S*. *aureus*. However, higher numbers of *APP* passed the filter a fact that so far remains unexplained.

It was found that Gram-negative bacteria experience stress during aerosolization by collision nebulizers [33], a fact that is confirmed by own preliminary tests using *E*. *coli* and *Pseudomonas aeruginosa* (see S2 Table). In the present study, viability of PRRSV and APP decreased substantially whereas BEV and *S*. *aureus* were more robust. Aerosolization stress influences culturability possibly resulting in false high reduction efficiencies. Reduction rates were calculated from aerosolized pathogens sampled in front of and behind the air filter. Hence, pathogens in these samples were likewise impaired by aerosolization. However, this does not fully exclude but diminishes possible calculation errors. Moreover, aerosolized bacteria carry electric charges that play an important role when particles are collected on filters [34]. Unfortunately, devices for charge neutralization were not available for our study.

Despite a high reduction rate for certain filter prototypes, viable (i.e. infectious) pathogens were recovered from the clean gas side in any experiment except for BEV. BEV is well known to be highly susceptible to inactivation by drying [35] which is confirmed by our investigations as live BEV was only occasionally cultured from samples taken in front of the filter during tests at a flow rate of 80 m^3^/h (prototype 4), but not in any sample from trials at the higher volume flow rate of 1,800 m^3^/h. For this reason, BEV analyses were carried out by quantitative real-time RT-PCR which proved to be a valid method.

Other studies mainly included experimental models or controlled field models and evaluated air filters regarding their ability to reduce aerosol transmission of PRRSV from infected donor pigs housed adjacent to naïve recipients [1,2,15,16,30]. Only one study [36] evaluated mechanical filters (MERV 14/EU F8 and MERV 16/EU F9) using PRRSV similar to the study presented here. Overall, our results regarding PRRSV are in concordance with this study although test chamber design, virus concentration (10^1^ to 10^7^ TCID_50_/l), air velocity (1.1 m/s), and diagnostic assays (RT-PCR, swine bioassay) differed from our study. In contrast to our study, one MERV 16 filter tested by our colleagues [36] was able to completely prevent the transport of airborne PRRSV [4].

With regard to biosafety precautions at filter change under field conditions, we further investigated viability and infectivity of the respective pathogen inside the filter material over time at room temperature. In addition, the question came up whether certain pathogens might be able to multiply or accumulate inside the filter matter hence representing a source of infection, too. Depending on the filter *S*. *aureus* was still viable four weeks after the experiment, whereas *APP* was no longer cultivable at 24 h after the experiment. *APP* has a short survival time on dry surfaces and even survived less than one day when held under natural conditions of humidity [37] whereas *S*. *aureus* has been described as resistant to desiccation [38] and consequently survives over a longer period on dry surfaces [39] which supports our results. PRRSV stayed infectious only in the secondary filter of prototype 1 and in the filter mat of prototype 4. In both, infectivity diminished after 24 h of storage. It was not recovered from the prefilter of prototype 1 maybe due to the structure of this coarse particle filter which might be unable to retain this small virus. Overall, this leads to the assumption that virus accumulation and multiplication of bacteria inside the filter materials is unlikely to occur. Nevertheless, as pathogens generally survived up to weeks, personal protective equipment should be used during change of filters.

Based on our findings and studies published elsewhere (e.g. [1,15,16]) it can be ascertained that air filtration combined with other strict biosecurity measures remarkably reduces the risk of introducing airborne transmitted pathogens to animal housings. To further evaluate the influence of air filtration systems on indoor air quality and animal health, air filtering systems based on our prototypes 1 and 4 were installed into a pig facility in Saxony, Germany. After three consecutive fattening periods results are currently under analysis with special emphasis on air quality and lung health of pigs.

## Conclusions

Depending on the filter matter and pathogen, a reduction rate of up to 3 log_10_-steps was achieved at laboratory scale. Filter efficiency was much higher for bacteria compared to viruses mainly due to filter structure and size of the respective pathogen. Despite a reduction of up to 99.9%, infectious particles passed all filters with the exception of BEV. However, the latter result must be attributed to the sensitivity of BEV against desiccation. Pathogen viability faded over storage time at room temperature, hence, an accumulation or multiplication of pathogens in the filter mat is unlikely; an important fact with regard to filter replacement by staff members. In concordance with other studies we clearly demonstrated the efficacy of air filters. However, more information on their efficacy in the field is needed before conclusions on animal health can be drawn.

## Supporting information

S1 Table Filter efficiency measured using different viruses. Raw data obtained from five replicates.
(PDF)

S2 Table Filter efficiency measured using different bacteria. Raw data obtained from five replicates.
(PDF)

S3 Table Kinetic of infectivity. (PDF)
