# Supplementary material for: Efficiency of different air filter types for pig facilities at laboratory scale
Source: PLoS One. 2017 Oct 13;12(10):e0186558. doi: 10.1371/journal.pone.0186558 (PMC5640248; doi:10.1371/journal.pone.0186558)
Supplement: S1 Table — Raw data obtained from five replicates. (PDF) [file pone.0186558.s001.pdf]

S1 Table. Filter efficiency measured using different viruses. Raw data obtained from five replicates.

Filter prototype	Trial	Pathogen	Volume flow rate (m^3/h)	Frequency converter	Pressure calibrator (Pa)	Atomizer gauze pressure (bar)	$TCID_{50}$/ml (each sample was analyzed twice)										Pathogen reduction (%)		Standard deviation (%)	Mean reduction efficiency (%) #	Copies/µl determined by quantitative real-time RT-PCR		Pathogen reduction (%)	Mean reduction efficiency (%)	Standard deviation (%)
							original culture suspension	original culture suspension	culture remnants retrieved from Atomizer bowl	culture remnants retrieved from Atomizer bowl	in front of the filter	in front of the filter	mean (in front of the filter)	behind the filter	behind the filter	Mean (behind the filter)					in front of the filter	behind the filter			
	1	EAV	1800	92%	163	5			$10^{6,7}$	$10^{7,0}$	$10^{3,4}$	nd		$10^{2,0}$	nd		96.02	n.d.							
	2	EAV	1800	92%	163	5			$10^{6,7}$	$10^{7,3}$	$10^{3,0}$	nd		$10^{1,8}$	nd		93.69	n.d.							
2	3	EAV	1800	92%	163	5	$10^{7,3}$	$10^{7,6}$	$10^{7,0}$	$10^{7,0}$	$10^{3,7}$	nd	$10^{3,6}$	$10^{2,4}$	nd	$10^{2,0}$	94.99	n.d.	2.36	97.48	nd	nd	nd	nd	nd
	4	EAV	1800	92%	163	5			$10^{6,3}$	$10^{6,3}$	$10^{4,4}$	nd		$10^{2,0}$	nd		99.60	n.d.							
	5	EAV	1800	92%	163	5			$10^{7,0}$	$10^{7,0}$	$10^{3,4}$	nd		$10^{1,7}$	nd		98.00	n.d.							
	1	EAV	1800	92%	163	5			$10^{7,0}$	$10^{7,3}$	$10^{4,0}$	$10^{3,3}$		$10^{2,6}$	$10^{1,7}$		96.02	97.49							
	2	EAV	1800	92%	163	5			$10^{7,3}$	$10^{7,3}$	$10^{3,6}$	$10^{3,6}$		$10^{2,0}$	$10^{1,7}$		97.49	98.74							
1	3	EAV	1800	92%	163	5	$10^{6,7}$	nd	$10^{8,0}$	$10^{7,6}$	$10^{3,7}$	$10^{4,0}$	$10^{3,7}$	$10^{2,0}$	$10^{2,6}$	$10^{2,1}$	98.00	96.02	1.19	97.49	nd	nd	nd	nd	nd
	4	EAV	1800	92%	163	5			$10^{7,3}$	$10^{7,6}$	$10^{3,7}$	$10^{3,7}$		$10^{2,0}$	$10^{2,0}$		98.00	98.00							
	5	EAV	1800	92%	163	5			$10^{7,7}$	$10^{7,0}$	$10^{3,6}$	$10^{4,0}$		$10^{2,3}$	$10^{2,3}$		94.99	98.00							
	1	EAV	80	49%	12.6	3.5			$10^{7,7}$	$10^{7,6}$	$10^{5,0}$	$10^{5,0}$		$10^{3,3}$	$10^{3,0}$		98.00	99.00							
	2	EAV	80	49%	12.6	3.5			$10^{7,3}$	$10^{8,0}$	$10^{4,7}$	$10^{4,7}$		$10^{4,0}$	$10^{4,0}$		80.05	80.05							
3	3	EAV	80	52%	12.6	3.5	$10^{7,6}$	$10^{7,6}$	$10^{7,6}$	$10^{7,7}$	$10^{5,3}$	$10^{4,7}$	$10^{5,0}$	$10^{3,7}$	$10^{3,3}$	$10^{3,6}$	97.49	96.02	13.01	96.02	nd	nd	nd	nd	nd
	4	EAV	80	57%	12.6	3.5			$10^{7,0}$	$10^{7,3}$	$10^{4,7}$	$10^{5,3}$		$10^{4,3}$	$10^{4,0}$		60.19	94.99							
	5	EAV	80	61%	12.6	3.5			$10^{8,0}$	$10^{8,3}$	$10^{5,3}$	$10^{5,6}$		$10^{3,3}$	$10^{3,0}$		99.00	99.75							
	1	EAV	80	29%	12.6	3.5			$10^{7,3}$	$10^{7,0}$	$10^{4,6}$	$10^{4,6}$		$10^{2,7}$	$10^{2,7}$		98.74	98.74							
	2	EAV	80	29%	12.6	3.5			$10^{7,6}$	$10^{8,0}$	$10^{4,7}$	$10^{4,7}$		$10^{3,3}$	$10^{3,3}$		96.02	96.02							
4	3	EAV	80	29%	12.6	3.5	$10^{7,3}$	$10^{7,3}$	$10^{7,6}$	$10^{8,0}$	$10^{4,6}$	$10^{4,7}$	$10^{4,7}$	$10^{2,7}$	$10^{2,7}$	$10^{2,8}$	98.74	99.00	1.26	98.74	nd	nd	nd	nd	nd
	4	EAV	80	29%	12.6	3.5			$10^{7,3}$	$10^{7,0}$	$10^{4,7}$	$10^{5,0}$		$10^{2,7}$	$10^{2,7}$		99.00	99.50							
	5	EAV	80	29%	12.6	3.5			$10^{7,0}$	$10^{7,6}$	$10^{4,3}$	$10^{5,0}$		$10^{2,3}$	$10^{3,0}$		99.00	99.00							
	1	PRRSV	1800	90%	163	5			nd	nd	$10^{3,65}$	$10^{3,9}$		$10^{1,9}$	$10^{1,975}$		97.89	99.00							
	2	PRRSV	1800	90%	163	5			nd	nd	$10^{4,3}$	$10^{3,7}$		$10^{2,3}$	$10^{2,3}$		99.00	95.53							
1	3	PRRSV	1800	90%	163	5	$10^{5,975}$	$10^{5,625}$	nd	nd	$10^{3,975}$	$10^{3,7}$	$10^{3,8}$	$10^{2,3}$	$10^{1,975}$	$10^{2,1}$	97.89	97.89	1.05	98.00	nd	nd	nd	nd	nd
	4	PRRSV	1800	90%	163	5			nd	nd	$10^{3,95}$	$10^{3,6}$		$10^{1,975}$	$10^{1,975}$		98.94	97.76							
	5	PRRSV	1800	90%	163	5			nd	nd	$10^{3,95}$	$10^{3,6}$		$10^{2,3}$	$10^{1,65}$		97.76	98.94							
	1	PRRSV	80	29%	12.6	3.5			$10^{5,7}$	$10^{6,3}$	$10^{2,7}$	$10^{3,0}$		$10^{1,7}$	$10^{1,7}$		90.00	94.99							
	2	PRRSV	80	29%	12.6	3.5			$10^{5,3}$	$10^{6,0}$	$10^{2,7}$	$10^{2,7}$		$10^{2,0}$	$10^{1,7}$		80.05	90.00							
4	3	PRRSV	80	29%	12.6	3.5	$10^{6,0}$	$10^{4,6}$	$10^{6,0}$	$10^{5,3}$	$10^{3,0}$	$10^{2,7}$	$10^{2,9}$	$10^{1,7}$	$10^{1,7}$	$10^{1,8}$	94.99	90.00	5.96	92.06	nd	nd	nd	nd	nd
	4	PRRSV	80	29%	12.6	3.5			$10^{6,0}$	$10^{5,6}$	$10^{3,0}$	$10^{3,0}$		$10^{1,7}$	$10^{1,7}$		94.99	94.99							
	5	PRRSV	80	29%	12.6	3.5			$10^{5,7}$	$10^{5,6}$	$10^{2,7}$	$10^{3,0}$		$10^{2,0}$	$10^{1,7}$		80.05	94.99							
	1	BEV-1	1800	89%	163	5			$10^{5,7}$	$10^{5,7}$	0	0		0	0		na	0.00			2.150E+06	1.950E+05	90.93		
	2	BEV-1	1800	89%	163	5			$10^{6,0}$	$10^{6,0}$	0	0		0	0		na	0.00			5.970E+06	1.340E+05	97.76		
1	3	BEV-1	1800	89%	163	5	$10^{5,3}$	$10^{5,0}$	$10^{6,0}$	$10^{5,3}$	0	0	0	0	0	0	na	0.00	na	na	9.460E+06	3.070E+05	96.75	95.98	2.90
	4	BEV-1	1800	89%	163	5			$10^{6,3}$	$10^{6,0}$	0	0		0	0		na	0.00			6.380E+06	2.300E+05	96.39		
	5	BEV-1	1800	89%	163	5			$10^{5,0}$	$10^{5,6}$	0	0		0	0		na	0.00			3.860E+06	7.520E+04	98.05		
	1	BEV-1	80	28%	12.6	3.5			$10^{5,65}$	$10^{4,975}$	$10^{0,7}$	0		0	0		na	0.00			8.890E+07	7.930E+05	99.11		
	2	BEV-1	80	28%	12.6	3.5			$10^{6,275}$	$10^{5,975}$	$10^{1,3}$	0		0	0		na	0.00			1.480E+08	1.780E+06	98.80		
4	3	BEV-1	80	28%	12.6	3.5	$10^{5,6}$	$10^{4,65}$	$10^{5,65}$	$10^{5,975}$	0	0	$10^{0.4}$	$10^{0,7}$	0	$10^{0.1}$	na	74.88	na	na	8.250E+07	2.120E+06	97.43	98.69	0.72
	4	BEV-1	80	28%	12.6	3.5			$10^{5,265}$	$10^{5,65}$	0	0		0	0		na	0.00			1.260E+08	1.190E+06	99.06		
	5	BEV-1	80	28%	12.6	3.5			$10^{5,265}$	$10^{5,975}$	10^{1}	$10^{0,7}$		0	0		na	0.00			1.540E+08	1.420E+06	99.08		

nd - not done, na - not applicable

(100 - (Mean behind the Filter x 100) : Mean in front of the filter))
