# Supplementary material for: Efficiency of different air filter types for pig facilities at laboratory scale
Source: PLoS One. 2017 Oct 13;12(10):e0186558. doi: 10.1371/journal.pone.0186558 (PMC5640248; doi:10.1371/journal.pone.0186558)
Supplement: S2 Table — Raw data obtained from five replicates. (PDF) [file pone.0186558.s002.pdf]

S2 Table. Filter efficiency measured using different bacteria. Raw data obtained from five replicates.

Filter prototype	Trial	Pathogen	Volume flow rate (m^3/h)	Pressure calibrator (Pa)	Atomizer gauze pressure (bar)	cfu/ml				Pathogen reduction (%)	Mean reduction efficiency (%)	Standard deviation (%)
						original culture suspension	culture remnants retrieved from Atomizer bowl	in front of the filter	behind the filter			
1	1	S. aureus DSM 799	1800	163	5	6.20E+08	9.00E+07	4.10E+05	6.32E+03	98.46	98.61	0.29
	2	S. aureus DSM 799	1800	163	5		6.90E+08	4.00E+05	7.23E+03	98.19		
	3	S. aureus DSM 799	1800	163	5		1.10E+09	6.00E+05	7.68E+03	98.72		
	4	S. aureus DSM 799	1800	163	5		1.50E+09	6.00E+05	7.55E+03	98.74		
	5	S. aureus DSM 799	1800	163	5		1.30E+09	6.70E+05	7.05E+03	98.95		
2	1	S. aureus DSM 799	1800	163	5	1.50E+08	1.40E+08	1.30E+05	1.18E+03	99.09	99.15	0.21
	2	S. aureus DSM 799	1800	163	5		9.70E+08	2.50E+05	2.82E+03	98.87		
	3	S. aureus DSM 799	1800	163	5		1.10E+09	4.10E+05	3.68E+03	99.10		
	4	S. aureus DSM 799	1800	163	5		1.40E+09	5.00E+05	2.91E+03	99.42		
	5	S. aureus DSM 799	1800	163	5		1.30E+09	3.00E+05	2.14E+03	99.29		
4	1	S. aureus DSM 799	80	12.6	3.5	1.30E+08	4.50E+05	8.20E+04	0.00E+00	100.00	99.97	0.07
	2	S. aureus DSM 799	80	12.6	3.5		4.50E+08	3.10E+05	4.55E+02	99.85		
	3	S. aureus DSM 799	80	12.6	3.5		8.70E+08	6.10E+05	0.00E+00	100.00		
	4	S. aureus DSM 799	80	12.6	3.5		6.80E+08	9.50E+05	0.00E+00	100.00		
	5	S. aureus DSM 799	80	12.6	3.5		5.80E+08	7.30E+05	0.00E+00	100.00		
1	1	APP DSM 13474	1800	163	5	6.10E+08	4.80E+07	8.60E+03	7.30E+02	91.51	95.21	3.34
	2	APP DSM 13474	1800	163	5			4.70E+03	5.90E+01	98.74		
	3	APP DSM 13474	1800	163	5			1.80E+02	9.10E+00	94.94		
	4	APP DSM 13474	1800	163	5			2.90E+02	4.50E+00	98.45		
	5	APP DSM 13474	1800	163	5	1.50E+08	1.00E+07	1.20E+02	9.10E+00	92.42		
4	1	APP DSM 13474	80	12.6	3.5	3.50E+08	4.60E+07	3.40E+04	4.10E+01	99.88	99.90	0.05
	2	APP DSM 13474	80	12.6	3.5			7.90E+04	7.30E+01	99.91		
	3	APP DSM 13474	80	12.6	3.5			7.60E+04	4.50E+01	99.94		
	4	APP DSM 13474	80	12.6	3.5			8.00E+04	4.50E+01	99.94		
	5	APP DSM 13474	80	12.6	3.5	3.40E+08	5.40E+07	6.40E+04	1.20E+02	99.81		
pretest without filter	1	M. hyorhinis DSM 25591	1800	163	5	1.30E+07	1.10E+06	0.00E+00	0.00E+00	na	na	na
	2	M. hyorhinis DSM 25591	1800	163	5	3.60E+06	3.50E+05	0.00E+00	0.00E+00	na		
pretest without filter	1	P. aeruginosa DSM 939	1800	163	5	6.80E+09	1.50E+09	0.00E+00	0.00E+00	na	na	na
	2	P. aeruginosa DSM 939	1800	163	5	9.00E+09	6.30E+08	2.50E+01	0.00E+00	nd		
	3	P. aeruginosa DSM 939	1800	163	5	9.50E+09	1.00E+09	0.00E+00	0.00E+00	na		
	4	P. aeruginosa DSM 939	1800	163	5	2.90E+09	1.00E+09	0.00E+00	0.00E+00	na		
	5	P. aeruginosa DSM 939	1800	163	5	2.30E+09	8.10E+08	0.00E+00	0.00E+00	na		
	6	P. aeruginosa DSM 939	1800	163	5	1.30E+09	2.00E+08	0.00E+00	0.00E+00	na		
	7	P. aeruginosa DSM 939	1800	163	5	1.70E+09	2.50E+08	0.00E+00	0.00E+00	na		
	8	P. aeruginosa DSM 939	1800	163	5	8.10E+08	1.80E+08	8.30E+00	0.00E+00	nd		
pretest without filter	1	E. coli DSM 682	1800	163	5	4.70E+09	2.90E+08	0.00E+00	0.00E+00	na	na	na
