# Supplementary material for: Efficiency of different air filter types for pig facilities at laboratory scale
Source: PLoS One. 2017 Oct 13;12(10):e0186558. doi: 10.1371/journal.pone.0186558 (PMC5640248; doi:10.1371/journal.pone.0186558)
Supplement: S3 Table — (PDF) [file pone.0186558.s003.pdf]

S3 Table. Kinetic of infectivity

Time of storage	Storage temperature (°C)	Filter prototype	Pathogen	Pathogen amount prefilter	Pathogen amount secondary filter
24 h	22		S. aureus DSM 799	150000 cfu/ml	4000 cfu/ml
48 h	22		S. aureus DSM 799	18000 cfu/ml	7200 cfu/ml
1 w	22	1	S. aureus DSM 799	5318 cfu/ml	814 cfu/ml
4 w	22		S. aureus DSM 799	80 cfu/ml	45 cfu/ml
8 w	22		S. aureus DSM 799	0 cfu/ml	0 cfu/ml
6 m	22		S. aureus DSM 799	71400 cfu/ml	9400 cfu/ml
24 h	22		S. aureus DSM 799	43000 cfu/ml	5000 cfu/ml
48 h	22		S. aureus DSM 799	600 cfu/ml	124 cfu/ml
1 w	22	2	S. aureus DSM 799	0 cfu/ml	0 cfu/ml
4 w	22		S. aureus DSM 799	nd	nd
8 w	22		S. aureus DSM 799		7360 cfu/ml
6 m	nd		S. aureus DSM 799		450 cfu/ml
24 h	22		S. aureus DSM 799		9.09 cfu/ml
48 h	22		S. aureus DSM 799		0 cfu/ml
7 d	22	4	S. aureus DSM 799		nd
4 w	22		S. aureus DSM 799	0 TCID50/ml	0 TCID50/ml
8 w	22		S. aureus DSM 799	0 TCID50/ml	0 TCID50/ml
6 m	nd		S. aureus DSM 799	0 TCID50/ml	0 TCID50/ml
30 min	22		BEV-1	0 TCID50/ml	0 TCID50/ml
60 min	22		BEV-1	0 TCID50/ml	0 TCID50/ml
2 h	22		BEV-1	0 TCID50/ml	0 TCID50/ml
4 h	22		BEV-1	nd	nd
24 h	22	1	BEV-1	nd	nd
48 h	22		BEV-1	nd	nd
1 w	nd		BEV-1		0 TCID50/ml
4 w	nd		BEV-1		0 TCID50/ml
6 m	nd		BEV-1		0 TCID50/ml
30 min	22		BEV-1		0 TCID50/ml
60 min	22		BEV-1		0 TCID50/ml
4 h	22		BEV-1		nd
24 h	22		BEV-1		nd
48 h	22	4	BEV-1		nd
1 w	nd		BEV-1		$10^{1.975}$ TCID50/ml
4 w	nd		BEV-1		0 TCID50/ml
6 m	nd		BEV-1		$10^{1.8}$ TCID50/ml
30 min	22		PRRSV		$10^{1.3}$ TCID50/ml
60 min	22		PRRSV		$10^{2.3}$ TCID50/ml
2 h	22		PRRSV		0 TCID50/ml
4 h	22		PRRSV		0 TCID50/ml
24 h	22	4	PRRSV		nd
48 h	22		PRRSV		nd
1 w	22		PRRSV	0 TCID50/ml	cpe, uncalculable
4 w	nd		PRRSV	0 TCID50/ml	$10^{1.975}$ TCID50/ml

6 m	nd		PRRSV	0 TCID50/ml	cpe, uncalculable
30 min	22		PRRSV	0 TCID50/ml	cpe, uncalculable
60 min	22		PRRSV	0 TCID50/ml	cpe, uncalculable
2 h	22		PRRSV	0 TCID50/ml	0 TCID50/ml
4 h	22		PRRSV	0 TCID50/ml	0 TCID50/ml
24 h	22	1	PRRSV	0 TCID50/ml	0 TCID50/ml
48 h	22		PRRSV	nd	nd
1 w	22		PRRSV	1100 cfu/ml	970 cfu/ml
4 w	22		PRRSV	1300 cfu/ml	150 cfu/ml
6 m	nd		PRRSV	400 cfu/ml	210 cfu/ml
30 min	22		APP DSM 13474	200 cfu/ml	40 cfu/ml
60 min	22		APP DSM 13474	0 cfu/ml	0 cfu/ml
2 h	22		APP DSM 13474	0 cfu/ml	0 cfu/ml
4 h	22		APP DSM 13474	0 cfu/ml	0 cfu/ml
24 h	22	1	APP DSM 13474	nd	nd
48 h	22		APP DSM 13474	nd	nd
1 w	22		APP DSM 13474	610 cfu/ml	
4 w	nd		APP DSM 13474	100 cfu/ml	
6 m	nd		APP DSM 13474	0 cfu/ml	
30 min	22		APP DSM 13474	32 cfu/ml	
60 min	22		APP DSM 13474	0 cfu/ml	
2 h	22		APP DSM 13474	0 cfu/ml	
4 h	22		APP DSM 13474	0 cfu/ml	
24 h	22	4	APP DSM 13474	nd	
48 h	22		APP DSM 13474	nd	
1 w	22		APP DSM 13474	0.00E+00	
4 w	nd		APP DSM 13474	nd	
6 m	nd		APP DSM 13474	nd	

w - weeks, m - months
nd - not done, cpe - cytopathogenic effect
